# Massive Myelomatous Pleural Effusion With Contralateral Mediastinal Shift: A Unique Presentation of Extramedullary Myeloma

**DOI:** 10.7759/cureus.43040

**Published:** 2023-08-06

**Authors:** Alexander G Polski, Jerome Ramirez Marquez, Samuel McQuiston

**Affiliations:** 1 Radiology, USA Health University Hospital, Mobile, USA

**Keywords:** pleural effusion cytology, tension, malignant pleural effusion, plasmacytoma, myeloma

## Abstract

Multiple myeloma (MM) is a relatively common malignancy that primarily affects the bone marrow, while extramedullary disease (EMD) occurs in the skin and muscle, lung pleura, lymph nodes, liver, and CNS. Myelomatous pleural effusion (MPE) is a rare extramedullary manifestation of MM in which pleural fluid is composed almost entirely of abnormal plasma cells. MPE and other types of EMD are associated with poor prognosis, and MPE can present emergently due to tension physiology. We report a case of a patient with massive MPE presenting with contralateral midline shift. There are exceedingly few such cases and this report highlights a unique presentation of this rare clinical entity. Epidemiology, radiographic features, diagnosis, treatment, and implications for the prognosis of the disease are discussed.

## Introduction

Multiple myeloma (MM) is a cytogenetically heterogeneous monoclonal plasma cell malignancy that primarily affects the skeletal system by proliferating in the bone marrow, producing lytic lesions in the affected portion of bone due to dysregulation of physiologic bone homeostatic mechanisms by neoplastic plasma cells. Hyperproduction of immunoglobulins by neoplastic plasma cells also contributes to the pathologic features of the condition. It is responsible for 1% of malignancies and 10% of hematologic malignancies [1].

In the United States, the mean age at diagnosis of MM is 69 years. Males are at 1.5 times the risk of females, and individuals of African descent are the highest risk demographic, at greater than two times the risk of individuals of European descent, with even greater risk when considering early onset presentation MM [2]. Clinical and laboratory findings include hypercalcemia, renal insufficiency, anemia, and bone lytic lesions (CRAB) features as well as amyloidosis. Initial labs include serum or urine protein electrophoresis or serum-free light chain analysis followed by bone marrow biopsy. Diagnosis involves evidence of ≥10% of clonal plasma cells on bone marrow biopsy or biopsy-proven plasmacytoma, and evidence of end-organ damage as noted above. In patients without CRAB features, diagnosis is made based on clonal cells ≥60% on bone marrow biopsy, serum free light chain ratio ≥100, or more than one focal lesion on CT or MRI [3].

Myelomatous pleural effusion (MPE) is a rare extramedullary manifestation of MM in which pleural fluid is composed almost entirely of abnormal plasma cells, occurring in <1% of cases of MM. Extramedullary disease (EMD) develops when a clonal line acquires the ability to proliferate outside of the medullary tumor microenvironment. EMD most commonly affects the integument and muscle, pleura, lymph nodes, liver, and CNS [4]. MPE is typically secondary to a pleural plasmacytoma, but can also occur due to extension of chest wall plasmacytoma, lymphatic obstruction from mediastinal lymph node involvement, and invasion from adjacent skeletal lesions [5]. MPE typically presents with symptoms related to the pleural effusion such as progressively worsening dyspnea, pleuritic chest pain, non-productive cough, and orthopnea but can include symptoms indicating hemodynamic compromise (tachycardia, tachypnea, hypotension, hypoxia) if tension physiology is present. Due to the dismal prognosis carried by MPE and the comparatively high proportion of EMD in relapse after allogeneic or autologous stem cell transplants, it is imperative for clinicians and diagnosticians to be aware of this manifestation of the disease [6]. 

Here, we present a case of a MM patient with EMD present on diagnosis, refractory to autologous stem cell transplant, and several lines of chemotherapy presenting with massive left-sided MPE with significant right-sided mediastinal shift.

## Case presentation

**Figure 1:**
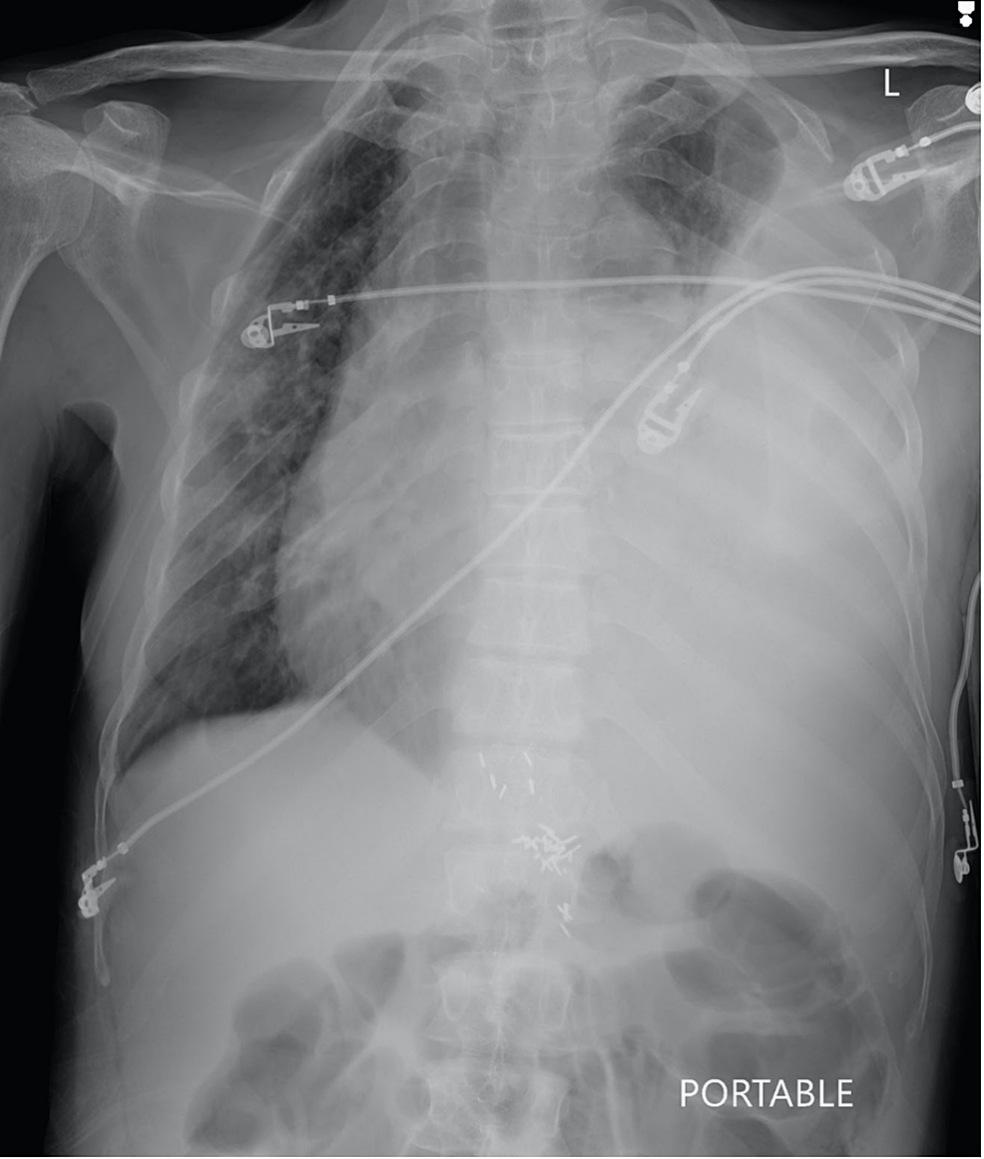
Chest x-ray demonstrating left-sided pleural effusion with rightward mediastinal shift consistent with a large-volume hydrothorax.

**Figure 2:**
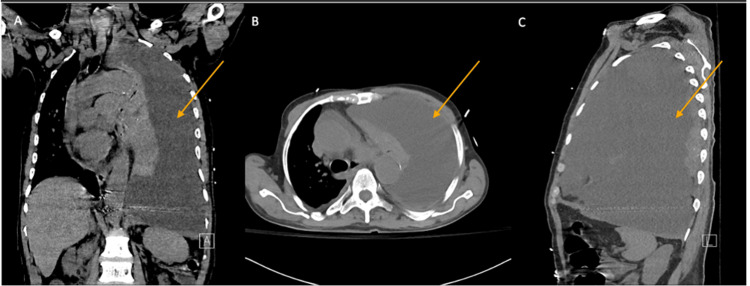
Chest CT demonstrating large left pleural effusion resulting in complete ipsilateral lung collapse.

A 67-year-old African American male with a past medical history of peptic ulcer disease, Paget’s disease, and MM status post-autologous stem cell transplant presented to the ED with worsening dyspnea. Review of systems identified non-productive cough, shortness of breath, fatigue, pleuritic chest pain, orthopnea, and general malaise. Upon presentation, the patient was tachypneic and hypoxic, requiring 3L O2 to maintain saturation above 94%. Physical exam was remarkable for absent breath sounds of the left lung fields and positive jugular venous distention. Chest x-ray revealed left-sided pleural effusion with rightward mediastinal shift (Figure 1). Chest CT demonstrated a large left pleural effusion resulting in complete lung collapse (Figure 2).

**Figure 3:**
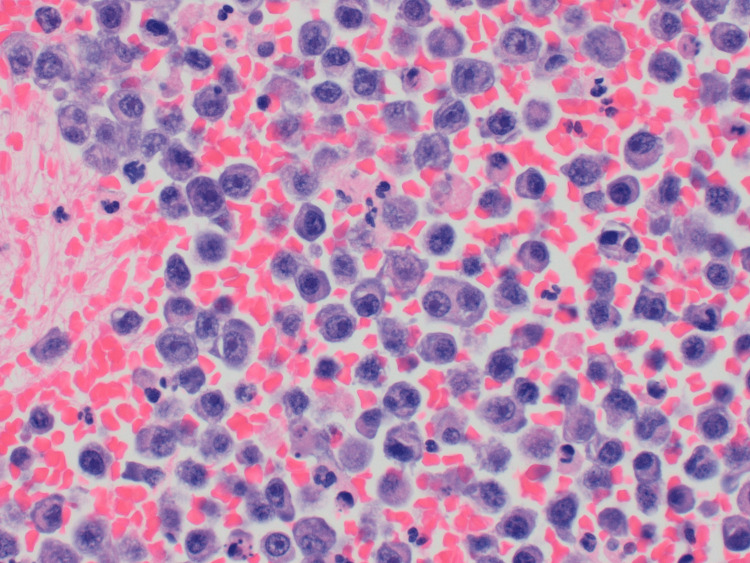
Numerous atypical large plasma cells with prominent nucleoli within effusion fluid. Hematoxylin & eosin stain magnification 400x.

An emergent thoracentesis was performed in the ED and the patient was admitted to the medical intensive care unit. The patient required two additional thoracenteses for persistent tension hydrothorax, resulting in a net volume of 3500cc removed from the pleural space. Fluid analysis performed with cytology confirmed an MPE consisting of anaplastic plasma cells (Figure 3). The patient’s subsequent hospital course was complicated by recurrent malignant pleural effusions with loculations, ultimately requiring the placement of a chest tube and treatment with intrapleural alteplase. His symptoms improved and the chest tube was removed. He was later discharged on palliative therapy with selinexor/dexamethasone for myeloma. The patient would later return with similar complaints but with worsening pancytopenia, fatigue, and renal function. He was subsequently admitted under hospice for end-of-life care given his complicated course with recurrent MPE and multiple comorbidities.

## Discussion

The incidence of EMD has been reported to be between 0.5-4.8% of new diagnoses of MM and 3.4-14% of relapsed cases [4,6]. Not counting paraosseous spread from focal skeletal lesions, the most common sites of EMD occurrence are integument and muscle (24%), pleura (12%), lymph nodes (10%), liver (9%), and the CNS (6%) [7]. Extramedullary MM of any type is associated with worse outcomes than MM with intramedullary disease only [8]. MPE in particular carries a significantly worse prognosis than other forms of extramedullary plasmacytoma, with the exception of CNS extramedullary plasmacytoma, which has the highest mortality [9]. Prior research indicates a median survival of approximately three to four months from the development of MPE [10,11]. Our patient had a survival of 49 days from the initial presentation. EMD can be present at diagnosis, as in this case, or it can develop later in the course. Patients with EMD at diagnosis have shorter overall survival than those who develop it after diagnosis or at relapse [9]. Patients who present with EMD also have a high rate of progression and relapse. 

Although up to 6% of cases of MM are associated with pleural effusion of any etiology, MPE occurs in <1% of all cases of MM [12,13] and, as in this case, is typically secondary to a pleural plasmacytoma [10]. Besides MPE, pleural effusions secondary to MM can be due to a number of etiologies such as heart failure due to amyloidosis, nephritic syndrome due to renal tubular paraprotein deposition and glomerular damage, and chronic renal failure [10]. Therefore, it should not be assumed that pleural effusion in a patient with MM is due to MPE. MPE is an exudative effusion and thus meets Light’s criteria (pleural protein/serum protein ratio >0.5 or pleural lactate dehydrogenase (LDH)/serum LDH radio >0.6), as opposed to the other common causes of myeloma-associated pleural effusion which are transudative in nature. CT attenuation values may be useful for the initial differentiation of transudative and exudative causes in a patient with MM, as transudative and exudative effusions have Hounsfield unit (HU) ranges between 2-15 and 4-33, respectively [14]. Exudative effusion should be considered if effusion attenuation is >15 HU, and although findings such as loculation or pleural thickening should raise suspicion for exudative cause, correlation with pleural fluid analysis is required as there is overlap in the attenuation ranges [14].

This case emphasizes the importance of clinical suspicion for MPE in a patient with a prior history of MM, in particular a history of stem cell transplantation, presenting with worsening dyspnea, pleuritic chest pain, non-productive cough, and orthopnea. Although acute management of MPE is no different from that of effusion of another etiology, it is imperative to assess pleural fluid for evidence of atypical plasma cells if MPE is suspected. Definitive diagnosis is made by cytological identification of atypical plasma cells or electrophoresis identification of gamma globulins within the effusion. 

While solitary pleural plasmacytoma in the absence of MM responds adequately to radiation therapy, a standard therapeutic guideline for the treatment of EMD in the setting of MM relapse does not exist. Typically, individualized, multimodal approaches combining radiation therapy, systemic immunomodulatory (thalidomide, pomalidomide, lenalidomide), and proteasome inhibitor (bortezomib, carfilzomib) therapies are utilized with varying degrees of success [15]. Use of adjuvant intrapleural chemotherapy has also been reported. 

It is imperative that any patient presenting with a pleural effusion be evaluated for signs suggestive of tension physiology. Clinically, these include tachycardia, hypotension, jugular venous distension, and cyanosis. Radiographic findings that should raise concern for tension physiology include contralateral tracheal/mediastinal shift and ipsilateral caudal displacement of the hemidiaphragm.

## Conclusions

We highlighted a case of massive MPE presenting as hydrothorax with a contralateral mediastinal shift in a patient with MM status post-chemotherapy and autologous stem cell transplant, including the initial presentation, diagnosis, and treatment course. Epidemiology, radiographic features, diagnosis, and prognosis were discussed for patients with MPE. This case illustrates the importance of clinical suspicion for MPE in patients with a history of MM presenting with worsening dyspnea, cough, and signs of hypoxia, and knowledge of the radiographic features of pleural effusion.
